# Plasma metabolomic analysis in mature female common bottlenose dolphins: profiling the characteristics of metabolites after overnight fasting by comparison with data in beagle dogs

**DOI:** 10.1038/s41598-018-30563-x

**Published:** 2018-08-13

**Authors:** Miwa Suzuki, Motoi Yoshioka, Yoshito Ohno, Yuichiro Akune

**Affiliations:** 1 Department of Marine Resources and Sciences, College of Bioresource Sciences, Nihon University, Kameino, Fujisawa, Kanagawa 252-0880 Japan; 2 Cetacean Research Center, Graduate School of Bioresources, Mie University, Kurimamachiya, Tsu, Mie 514-8507 Japan; 3 Port of Nagoya Public Aquarium, Minato, Nagoya, Aichi 455-0033 Japan

## Abstract

The present study was aimed at determining the characteristics of plasma metabolites in bottlenose dolphins to provide a greater understanding of their metabolism and to obtain information for the health management of cetaceans. Capillary electrophoresis-time-of-flight mass spectrometry (CE-TOFMS) and liquid chromatograph-time-of-flight mass spectrometry (LC-TOFMS) were conducted on plasma samples after overnight fasting from three common bottlenose dolphins as well as three beagle dogs (representative terrestrial carnivores) for comparison. In total, 257 and 227 plasma metabolites were identified in the dolphins and the dogs, respectively. Although a small number of animals were used for each species, the heatmap patterns, a principal component analysis and a cluster analysis confirmed that the composition of metabolites could be segregated from each other. Of 257 compounds detected in dolphin plasma, 24 compounds including branched amino acids, creatinine, urea, and methylhistidine were more abundant than in dogs; 26 compounds including long-chained acyl-carnitines and fatty acids, astaxanthin, and pantothenic acid were detected only in dolphins. In contrast, 25 compounds containing lactic acid and glycerol 3-phosphate were lower in dolphins compared to dogs. These data imply active protein metabolism, differences in usage of lipids, a unique urea cycle, and a low activity of the glycolytic pathway in dolphins.

## Introduction

Cetaceans are carnivorous mammals living in aquatic environments. To overcome the difficulties associated with an aquatic lifestyle, cetaceans have evolved a range of physiological and morphological modifications^1^. For example, adjustment to a marine environment is associated with the development of an extremely thick subcutaneous adipose layer (up to 50% of the body mass)^2^, formation of massive skeletal muscles^3^, a unique system for blood glucose balance^4^, a specialized osmoregulatory system^5^, and a diving response^6^. These specializations may influence the metabolism of dolphins by controlling cellular processes in response to various stimuli, nutritional conditions, and internal status.

The metabolic status of animals is generally monitored by quantitative analyses of substances in cells, tissues, and bio-fluids. Circulating blood includes molecules secreted or removed from tissues in response to physiological requirements; accordingly, the composition of plasma/serum can vary depending on the physiological status of the animal. In addition, pathological condition in tissues can lead to changes in blood composition. The development of a range of metabolomic platforms and technologies now permit the accurate assessment of a comprehensive set of metabolites in serum/plasma^7^. For health management of captive cetacean species, fundamental information on plasma metabolites will aid understanding of the characteristics of their metabolism, help in monitoring their health status, and be of value for identifying the causes of any health problems.

In a previous study, plasma amino acids of some dolphin species were analyzed using an automated amino acid analyzer and the results compared to those of mice^8^. It was found that circulating levels of carnosine, 3-methylhistidine, and urea were higher in dolphins than mice. More recently, a metabolomic platform for analysis of metabolites in exhaled breath of dolphins has been used^9,10^. However, a comprehensive analysis of circulating metabolites has not been reported so far in cetaceans. Thus, in the present study, we sought to characterize plasma metabolites in the common bottlenose dolphin through use of high-throughput analyses comparing with simultaneously-analyzed data from beagle dogs, a representative model animal for terrestrial carnivores, giving care of exclusion of the influential factors as possible^11^.

## Results and Discussion

### Detected metabolites

In the analysis of plasma metabolomics after overnight fasting in common bottlenose dolphin and beagle dog, a total of 297 peaks were determined as candidate compounds by CE-TOFMS and LC-TOFMS: 170 hydrosoluble metabolites (cation 114, anion 56) by CE-TOFMS and 127 lipophilic metabolites (positive 52, negative 75) by LC-TOFMS. The compounds detected in each species are listed in Table S2. In dolphin plasma, a total of 257 compounds were detected, while 241 were detected in the dogs. The number of compound detected were therefore similar in the two analyzed species; they were also similar to the reported previously for human plasma^12^, and slightly larger than that the 227 compounds detected in the plasma of northern elephant seal (*Mirounga angustirostris*), the only marine mammal species reported to date^13,14^. However, the differences in the contents of metabolites among species could not be compared because the reported data from previous studies did not include the necessary details. Some of the plasma metabolites detected in the present study, including amino acids and lipids, were also detected in an analysis of the exhaled breath of dolphins as nonvolatile compounds^9^ but most plasma metabolites were not detected.

**Figure 1:**
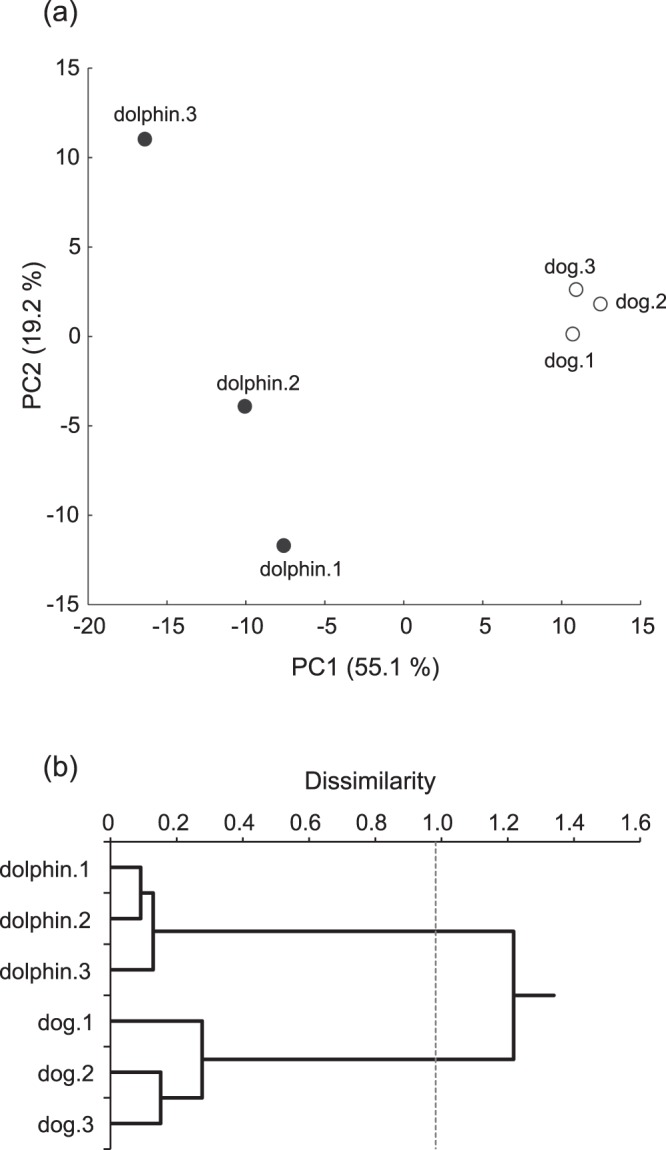
Similarity analyses of the plasma metabolites in common bottlenose dolphins and beagle dogs. (**a**) A principal component analysis score plots of the plasma metabolites in the dolphins (n = 3, black circles) and the dogs (n = 3, white circles). PC1 and PC2 are plotted on x- and y-axes, respectively. (**b**) A dendrogram of the plasma metabolites in the dolphins and the dogs. The x-axis shows the calculated distance between samples. The broken line indicates the branching point of the two clusters.

**Figure 2:**
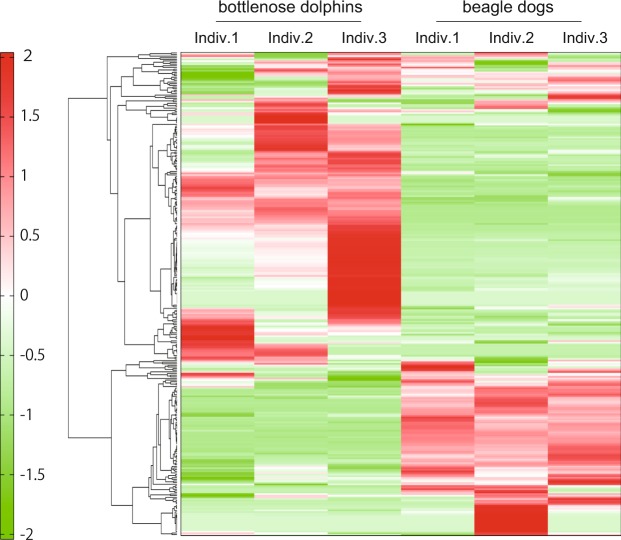
A heatmap of the plasma metabolomics data in common bottlenose dolphins (n = 3) and beagle dogs (n = 3). Hierarchical clustering was performed on standardized concentrations of each peak using average linkage. Each row represents one metabolite, and each column represents each sample. Darker green indicates the level of each peak is lower than the average, and darker red suggests a higher level than the average.

As shown in Fig. 1a, the PCA of peak data from dolphins and dogs identified significant differences among the components of plasma metabolites in the two species, with a larger deviation in dolphins than dogs. A cluster analysis using Ward’s method on the data for 250 metabolites in the individual samples resulted in two clusters according to species with commensurate intra-species similarities between the species (Fig. 1b). A heatmap of the data also showed different color patterns between the species with intra-species similarities (Fig. 2). The details of differences between species are described below. These results strongly suggest that plasma components after overnight fasting in dolphins differ from those of the dogs, possibly reflecting differences in metabolic status, with the possibility of influence of food content, despite the fact that both species are carnivorous mammals.

### Features of plasma metabolites in common bottlenose dolphin

The plasma metabolites showing significant differences in peak areas between dolphins and dogs are listed in Table 1. Despite using only three animals for each species, significant differences in abundance between species were detected in some metabolites; twenty-four compounds containing 10 amino acids or organic acids were more abundant in common bottlenose dolphins, and 25 compounds were more abundant in beagle dogs. Compounds detected in only one or other species are listed in Table 2. Twenty-six compounds, including many lipids, were detected only in dolphins, and 23 compounds were observed only in dogs.

**Table 1:** Metabolites in plasma with difference in relative area of peaks between common bottlenose dolphin and beagle dog (n = 3).

Compound name	KEGG ID	Bottlenose dolphin		Beagle dog		Dolphin vs beagle dog		
		Mean	S.D.	Mean	S.D.	Ratio	p-value	
**dolphins** > **dogs**								
1-Methylhistidine	No ID	1.4E-01	2.5E-02	1.8E-03	4.9E-04	78	0.010	*
3-Methylhistidine	C01152	3.2E-04	1.9E-05	1.1E-04	2.7E-05	2.8	7.6E-04	***
2-Hydroxyvaleric acid	No ID	3.2E-04	1.9E-05	1.1E-04	2.7E-05	2.8	7.6E-04	***
3-Methoxytyrosine	No ID	7.7E-03	9.3E-04	5.0E-03	1.1E-04	1.6	0.034	*
4-Methyl-2-oxovaleric acid 3-Methyl-2-oxovaleric acid	C00233 C00671	7.7E-03	9.3E-04	5.0E-03	1.1E-04	1.6	0.034	*
5-Methoxyindoleacetic acid	C05660	6.4E-04	4.9E-05	5.3E-04	2.2E-05	1.2	0.048	*
AC (20:0)	No ID	4.6E-06	9.2E-07	1.1E-06	1.3E-07	4.3	0.019	*
Anserine_divalent	C01262	6.7E-03	2.0E-03	9.9E-04	2.7E-04	6.8	0.037	*
Cholesterol sulfate	C18043	3.7E-04	1.0E-04	1.6E-05	3.8E-06	24	0.026	*
Creatine	C00300	3.4E-02	1.3E-03	6.9E-03	2.1E-03	5.0	1.8E-04	***
Cystine	C00491	6.5E-03	2.6E-04	5.4E-03	4.0E-04	1.2	0.021	*
Hecogenin	C08902	7.7E-05	1.3E-05	1.3E-05	1.5E-06	6.0	0.012	*
Hypoxanthine	C00262	1.3E-03	1.2E-04	5.3E-05	5.0E-06	25	0.003	**
Ile	C00407	2.8E-02	3.5E-03	1.9E-02	7.7E-04	1.4	0.047	*
Leu	C00123	6.0E-02	8.2E-03	4.2E-02	3.5E-03	1.4	0.047	*
Palmitoylcarnitine	C02990	2.5E-04	4.3E-05	7.8E-05	1.7E-05	3.3	0.011	*
Phe	C00079	3.6E-02	4.2E-03	2.1E-02	1.7E-03	1.7	0.016	*
Pipecolic acid	C00408	6.6E-04	2.1E-04	1.3E-04	7.1E-06	5.2	0.047	*
Taurochenodeoxycholic acid	C05465	2.1E-04	7.2E-05	4.3E-06	3.9E-06	49	0.039	*
Tyr	C00082	1.3E-02	2.3E-04	9.3E-03	5.8E-04	1.4	0.005	**
Urea	C00086	8.5E-01	9.3E-02	2.0E-01	4.2E-02	4.3	0.002	**
Val	C00183	7.6E-02	8.3E-03	4.7E-02	2.8E-03	1.6	0.018	*
β-Ala	C00099	7.0E-04	1.6E-04	1.7E-04	8.7E-06	4.1	0.028	*
**dolphins** < **dogs**								
1-Methylnicotinamide	C02918	3.7E-05	1.3E-05	2.6E-04	8.1E-05	0.14	0.039	*
2′-Deoxycytidine	C00881	6.3E-05	7.5E-06	2.5E-04	4.1E-05	0.3	0.014	*
3-Aminobutyric acid	No ID	8.5E-05	8.6E-06	1.5E-04	1.1E-05	0.6	0.001	**
3-Indoxylsulfuric acid	No ID	4.1E-04	2.2E-04	1.9E-03	4.9E-04	0.2	0.019	*
Betaine	C00719	1.3E-02	1.1E-03	7.5E-02	4.6E-03	0.2	0.001	**
Carnosine	C00386	5.6E-04	2.6E-04	1.9E-03	2.6E-04	0.3	0.003	**
Cystathionine	C00542	2.0E-04	4.0E-05	5.6E-04	1.5E-04	0.3	0.045	*
Cysteine glutathione disulfide	C05526	3.6E-05	1.1E-05	4.9E-04	3.4E-05	0.07	7.9E-04	***
Gln	C00064	9.5E-02	1.2E-02	1.4E-01	9.2E-04	0.7	0.024	*
His	C00135	1.6E-02	1.8E-03	2.3E-02	1.1E-03	0.7	0.009	**
Lactic acid	C00186	3.0E-02	2.1E-02	7.6E-02	1.6E-02	0.4	0.046	*
Linoleic acid	C01595	4.6E-04	4.0E-04	1.9E-03	3.8E-04	0.2	0.010	**
Lys	C00047	2.0E-02	2.3E-03	2.8E-02	1.5E-03	0.7	0.015	*
Malic acid	C00149	3.1E-04	5.7E-05	7.5E-04	1.2E-04	0.4	0.011	*
Met	C00073	7.3E-03	5.6E-04	1.3E-02	1.9E-03	0.5	0.025	*
N,N-Dimethylglycine	C01026	3.2E-04	6.9E-05	2.0E-03	3.8E-04	0.2	0.015	*
N5-Ethylglutamine	C01047	1.2E-03	2.0E-04	6.2E-03	1.1E-03	0.2	0.013	*
Penicillamine	C07418	1.0E-04	1.5E-05	2.1E-04	2.4E-05	0.5	0.006	**
Retinol-2	No ID	4.3E-06	2.0E-06	6.7E-05	2.8E-06	0.06	1.6E-05	***
Ricinoleic acid	C08365	5.1E-06	4.2E-06	6.9E-05	1.5E-05	0.07	0.012	*
S-Methylcysteine	No ID	1.4E-04	1.9E-05	4.9E-04	7.1E-05	0.3	0.009	**
Taurine	C00245	3.4E-03	3.7E-04	6.6E-03	2.9E-04	0.5	4.3E-04	***
Thr	C00188	2.3E-02	1.0E-03	4.6E-02	1.7E-03	0.5	1.5E-04	***
Trigonelline	C01004	4.9E-05	1.9E-05	5.2E-04	1.8E-04	0.09	0.043	*
Uric acid	C00366	4.0E-04	1.4E-04	8.1E-04	1.2E-04	0.5	0.020	*

*p < 0.05, **p < 0.01, ***p < 0.001.

**Table 2:** Metabolites detected in plasma of either common bottlenose dolphin or beagle dog.

Compound name	KEGG ID	Bottlenose dolphin		Beagle dog	
		Mean	S.D.	Mean	S.D.
1-Aminocyclopentanecarboxylic acid	C03969	9.7E-04	4.0E-04	N.D.	N.A.
1-Methyl-4-imidazoleacetic acid	C05828	4.0E-04	2.9E-04	N.D.	N.A.
3-Hydroxytetradecanoic acid	No ID	2.6E-06	1.2E-06	N.D.	N.A.
AC (12:0)	No ID	1.3E-05	3.7E-06	N.D.	N.A.
AC (16:1)	No ID	4.1E-06	9.1E-07	N.D.	N.A.
AC (20:1)	No ID	4.4E-06	3.3E-06	N.D.	N.A.
AC (22:0)	No ID	1.6E-06	2.9E-07	N.D.	N.A.
Astaxanthin	C08580	2.1E-06	1.4E-06	N.D.	N.A.
Azetidine 2-carboxylic acid	C08267	1.8E-04	1.2E-04	N.D.	N.A.
FA (13:0)	No ID	4.9E-05	2.4E-05	N.D.	N.A.
FA (14:1)-2-1	No ID	7.6E-06	6.6E-06	N.D.	N.A.
FA (15:1)-1-2 FA (15:1)-2-2	No ID No ID	8.6E-06	5.2E-06	N.D.	N.A.
FA (16:3)-2	No ID	1.8E-05	1.6E-05	N.D.	N.A.
FA (17:0)-1	No ID	1.9E-06	8.8E-07	N.D.	N.A.
FA (19:2)	No ID	4.4E-06	4.2E-06	N.D.	N.A.
FA (24:5)	No ID	9.1E-06	6.6E-06	N.D.	N.A.
FA (26:2)	No ID	7.2E-06	4.4E-06	N.D.	N.A.
Glycocholic acid	C01921	9.3E-07	4.2E-07	N.D.	N.A.
Guanidinosuccinic acid	C03139	2.1E-04	4.6E-05	N.D.	N.A.
Guanine	C00242	1.5E-04	3.3E-05	N.D.	N.A.
Hippuric acid	C01586	1.9E-04	3.5E-05	N.D.	N.A.
Homocystine	C01817	1.1E-04	1.7E-05	N.D.	N.A.
*N*-Acetyl-β-alanine	C01073	3.9E-04	9.4E-05	N.D.	N.A.
Pantothenic acid	C00864	2.2E-04	6.3E-05	N.D.	N.A.
Thiamine	C00378	4.5E-05	2.7E-05	N.D.	N.A.
15(S)-HETE	C04742	N.D.	N.A.	1.7E-06	5.2E-07
1*H*-Imidazole-4-propionic acid	No ID	N.D.	N.A.	1.8E-04	4.5E-05
2-Quinolinecarboxylic acid	C06325	N.D.	N.A.	3.7E-04	2.3E-04
5-Oxo-2-tetrahydrofurancarboxylic acid	No ID	N.D.	N.A.	2.6E-04	1.5E-05
Campesterol	C01789	N.D.	N.A.	4.8E-06	1.4E-06
Deoxycholic acid	C04483	N.D.	N.A.	1.5E-05	1.5E-05
FA (25:3)	No ID	N.D.	N.A.	3.4E-06	9.1E-07
Gluconic acid	C00257	N.D.	N.A.	3.9E-04	7.9E-05
Glutathione (GSSG)_divalent	C00127	N.D.	N.A.	2.0E-04	1.4E-05
Glycerol 3-phosphate	C00093	N.D.	N.A.	1.6E-04	3.1E-05
Indole-3-carboxaldehyde	C08493	N.D.	N.A.	6.4E-06	8.4E-07
Isovalerylalanine *N*-Acetylleucine	No ID C02710	N.D.	N.A.	2.1E-04	7.5E-05
Kynurenic acid	C01717	N.D.	N.A.	2.2E-05	1.8E-05
*N*-Acetylglutamine	No ID	N.D.	N.A.	1.6E-04	2.6E-05
*N*-Acetylhistidine	C02997	N.D.	N.A.	6.5E-05	1.5E-05
*p*-Hydroxymandelic acid	C03198	N.D.	N.A.	1.2E-04	1.9E-05
Phenaceturic acid	C05598	N.D.	N.A.	1.2E-04	2.4E-05
Retinol-1	No ID	N.D.	N.A.	1.3E-05	1.9E-06
*S*-Methylmethionine	C03172	N.D.	N.A.	5.1E-05	4.2E-06
Sitosterol	C01753	N.D.	N.A.	2.8E-06	9.0E-07
Stigmasterol	C05442	N.D.	N.A.	4.0E-06	1.2E-06
Urocanic acid	C00785	N.D.	N.A.	7.6E-05	7.7E-06

**Figure 3:**
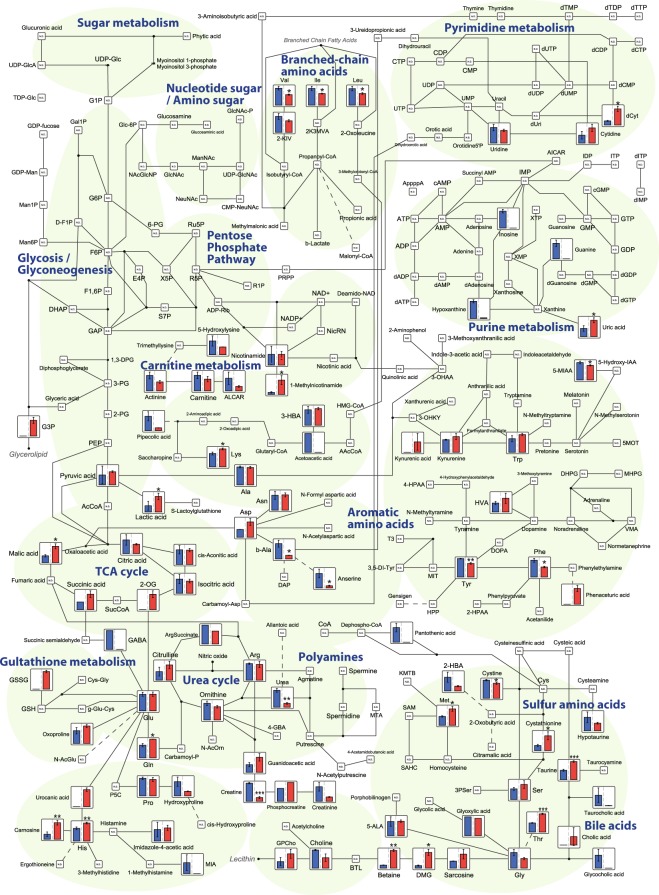
A metabolic pathway map in the plasma comparing common bottlenose dolphins (n = 3, blue columns) and beagle dogs (n = 3, red columns). Asterisks indicate significant differences in the peak areas of each plasma metabolite between animals (*p < 0.05, **p < 0.01, ***p < 0.001, Welch’s t-test).

A metabolic pathway map based on the plasma metabolomics data and a KEGG database analysis is shown in Fig. 3; this map visualizes the relative peak areas of each metabolite. As only molecules that are secreted, excreted, or discarded from cells circulate in the blood, then only some of the components of a pathway map are detected by plasma metabolomic analyses. In the following sections, we consider some of the metabolites that showed differences in abundance between the species.

### Nitrogen compounds

The relative levels of branched-chain amino acids (BCAAs), namely leucine (Leu) isoleucine (Ile) and valine (Val), were greater in dolphin plasma than in dog plasma (Table 1). In human plasma, BCAAs account for 40% of all essential amino acids^15^ and are metabolized mainly in skeletal muscle^16^. BCAAs play important roles for maintenance and recovery from exhaustion of skeletal muscle as part of the energy cycle and through use as components in protein synthesis^17^. The high levels of BCAAs in dolphin plasma suggest active muscular metabolism, especially catabolism. In addition, the abundance of BCAAs after overnight fasting may be due to “an insulin resistance-like symptom” known to be easily induced by short fasting in dolphins to maintain blood glucose levels^4^; it is known that circulating BCAAs are increased in type-2 diabetes patients with insulin resistance^18,19^.

Creatine was five-fold more abundant in dolphin plasma than dog plasma (Table 1). In addition, creatinine, an end product of creatine metabolism, was also 3.4-fold higher in dolphin, although the difference was on the cusp of significance (p = 0.052, Table S2). Creatine functions in skeletal muscle as a storage material with high-energy phosphate bonds^20^. A high level of creatine may be required to maintain the activity of skeletal muscle in dolphins.

The level of urea, an end product of protein metabolism, was 4.3-fold higher in dolphins than dogs (Table 1). Higher levels of urea and of urea nitrogen in blood indicate a more active protein metabolism in dolphins than in dogs^8,21^. In addition, active reabsorption of urea by the kidney to produce concentrated urine^5,22^ may contribute to its abundance in dolphin. Guanidinosuccinic acid was detected only in dolphin (Table 2). In uremic patients, guanidinosuccinate is synthesized in the liver from L-arginine and aspartic acid by an inhibitory effect due to accumulation of urea disrupting enzymes in the urea cycle^23^. A high concentration of circulating urea in dolphin could be related to the accumulation of guanidinosuccinic acid through changes in the urea cycle pathway, in a similar manner as uremia patients, as suggested previously^8^.

3-Methylhistidine was 2.8-fold more abundant in dolphin than dog (Table 1). This compound is mainly derived by methylation of histidine in actin and myosin in the muscle^24^; it is also a component of balenine, a dipeptide rich in whale muscle^25^. 3-Methylhistidine is used as an indicator of muscle catabolism, because it is directly excreted in urine and not reused for protein synthesis^26^. These data suggest active metabolism in the skeletal muscle of dolphin. The level of 1-methylhistidine that is a component of anserine (b-alanyl-1-methylhistidine) was 78-fold higher in dolphin than dog (Table 1). As β-alanine and anserine-divalent were also richer in dolphin plasma, we suggest that dolphin muscle is rich in anserine. By contrast, carnosine (β-alanyl-histidine; dipeptide) was more abundant in dog plasma. This may be the result of differences in the food of dogs and dolphins: meat-based diets are relatively richer in carnosine than fish-based diets^27,28^.

Phenylalanine (Phe) and tyrosine (Tyr) are also used as indicators of skeletal muscle degradation^29^; thus, the higher levels of these aromatic amino acids in dolphin (Table 1) is consistent with the higher active muscular metabolism indicated by the abundance of 3-methylhistidine. In addition, as Phe and Tyr are precursors of the catecholamines responsible for vascular control, which is vital for thermoregulation and diving responses in cetaceans^30–32^, high levels of these amino acids may be required for the underwater activities of dolphins.

The level of hypoxanthine was 25-fold higher in dolphin than dog (Table 1). Hypoxanthine is an ATP metabolite as well as an anaerobic metabolite; measurement of hypoxanthine in extracellular fluids provides information on changes in intracellular ATP, because a decrease in cellular ATP leads to a rise in the extracellular hypoxanthine level^33^. On the other hand, xanthine was not detected in dolphin, and uric acid was more abundant in dog than in dolphin. Xanthine and uric acid are derived from oxidation of hypoxanthine by perfusion after ischemia^34^. These results suggest that ATP degradation may occur by ischemia during diving, but that oxidative stress after reperfusion, leading to an increase in xanthine and uric acid, may not be excessive in the dolphin body.

### Lipids

The level of acylcarnitine (AC) (20:0) was higher in dolphin than dog (Table 1); moreover, several lipids such as acylcarnitines (AC 12:0, 16:1, 21:1 and 22:0) and fatty acids (FA 13:0, 14:1-2-1, 15:1-1-2, 15:1-2-1, 16:3-2, 17:0-1, 19:2, 24:5 and 26:2), were detected only in dolphin plasma (Table 2). The abundance of these lipids strongly suggests that dolphins can absorb and utilize the lipids from fish or may have a more complicated metabolic pathway for lipids than dogs to utilize them in various applications such as an energy resource, buoyancy material, and heat insulation. Recent reports have suggested the presence of a unique lipid metabolism in cetaceans^35,36^. Long-chain fatty acids (carbon number >12) generally combine with carnitine and exist as AC in the muscle, which allows them to pass through the mitochondrial inner membrane for β-oxidation and leads to ATP production^37^. Thus, dolphins could use lipids as a fuel for muscle activity more than dogs. The level of cholesterol sulfate was 24-fold higher in dolphin than in dog (Table 1). Much of the cholesterol sulfate may be of epidermal origin; a significant amount of cholesterol sulfate is produced in the epidermis in humans^38^ and the epidermis of cetaceans contains some cholesterol and its sulfate^39,40^.

### Glycolytic metabolism-related substances

Lactic acid was more abundant in dog plasma than dolphin plasma (Table 1). During intense activity of muscles, lactic acid is produced from glycogen and enters into circulation as lactate and is then removed; thus, if the supply/demand of glycogen and lactic acid is imbalanced, the circulating levels will be altered^41^. The lower level of lactic acid in dolphin may be due to a reduced dependence on carbohydrates for muscle energy production^42,43^. Glycerol 3-phosphate (G3P) was detected only in the dogs. This compound is a phosphoric ester of glycerol and is an intermediate in the glycolysis metabolic pathway^44^. The absence of G3P in dolphin plasma also suggests a lower activity of the glycolysis metabolic pathway.

### Other substances

Astaxanthin was detected only in dolphin. It is a xanthophyll carotenoid found in aquatic animals^45^ and exerts beneficial effects such as acting as an antioxidant^46^. As animals cannot synthesize astaxanthin, it is likely derived from their diet.

Pantothenic acid and thiamine were detected only in dolphin. Pantothenic acid is an essential component of coenzyme A (CoA) that plays a central role in the tricarboxylic acid cycle and in the synthesis of fatty acid as acetyl CoA or acyl CoA. Pantothenic acid also functions as a phosphopantetheine that is a cofactor of acyl carrier proteins involved in fatty acid metabolism^47^. The level of pantothenic acid in the blood is generally quite low; thiamine supplements increase the circulating levels of the compound^48^. Thus, the presence of thiamine may affect the level of circulating pantothenic acid. Thiamine also plays a role also as a co-enzyme and functions in metabolism of BCAAs and carbohydrates^49,50^. A high level of thiamine in dolphin plasma suggests it may be required for active BCAA metabolism in this species.

Taurochenodeoxycholic acid is one of the main bioactive substances in the bile and acts as a detergent to solubilize fats in the intestine^51^. The level of taurochenodeoxycholic acid was 49-fold higher in dolphin than in dog. This acid is secreted into the small intestine and absorbed by active transport in the intestine^51^. Glycocholic acid is a bile acid that emulsifies fats; it was detected only in dolphin plasma. These bile acids are absorbed in the small intestine and recirculated, and have many functions in metabolism^51,52^. The abundance of these acids in dolphin suggests that they are actively biosynthesized, secreted, and absorbed for the absorption of fats. By contrast, deoxycholic acid, a secondary bile acid synthesized by intestinal bacteria^53^, was detected only in dog. Other metabolites related to intestinal bacteria including 2-hydroxyvaleric acid^54^ and hecogenin^55^ were detected in dolphin at higher levels than in dog. These data suggest a difference in the components of intestinal bacteria between the species. Plant origin compounds including S-methylcysteine, campesterol, indole-3-carboxaldehyde, sitosterol, and stigmasterol were detected only in dog. These chemicals are possibly derived from enterobacteria or from the food provided to the dogs. Further studies are needed to determine the effects of enterobacteria on the metabolism of dolphins.

### Limitations

The principal limitation of this study is that plasma samples from only three animals of each species were used. The comparison of data from small numbers of animals obviously only allows unambiguous identification of conspicuous differences between the species; nevertheless, the comparison does allow characterization of plasma metabolites specific to dolphins. Welch’s t-test is widely applied to detect significant differences in data and is a reliable test when two samples have unequal variances^56^; thus, it is generally used for comprehensive analyses like metabolomic data analyses. However, Student’s t-test is more appropriate for small sample sizes (N ≤ 5) as Welch’s t-test has a greater risk of errors for small sample sizes^57^. Larger sample sizes would be needed to overcome this difficulty. However, the PCA on peak data from dolphins and dogs that identified significant differences among the components of plasma metabolites in the two species was supported by a cluster analysis using Ward’s method; the latter analysis yielded two clusters according to species with commensurate intra-species similarities in the species. These analyses reduce the shortcoming of the small sample sizes. The lack of data on non-fasted animals is another limitation in this study. It would be of value to investigated changes in metabolites before and after feeding to determine the complete characteristics of dolphin metabolism.

## Conclusion

Plasma metabolomic analysis using a combination of CE-TOFMS and LC-TOFMS in healthy, young mature female common bottlenose dolphins and beagle dogs after overnight fasting resulted in the detection of 257 and 227 plasma metabolites, respectively. The data showed that the composition of plasma metabolites was clearly different in the two species. The increased abundance of nitrogen compounds in dolphin, including BCAAs, creatinine, urea, and methylhistidine, suggest a higher level of active muscular metabolism in the aquatic animal. The variety and abundance of lipids including acylcarnitines and fatty acids and the high levels of some bile acids suggest that the dolphins have a more active and complicated metabolic pathway for lipids. On the other hand, the lower levels of lactic acid and G3P suggest a lower carbohydrate metabolic activity. Our data provide new insights into the adaptive physiological and metabolic changes in dolphins and also provide basic information for future studies on plasma/serum metabolomics in cetaceans.

## Methods

### Experimental ethics

This study was conducted in accordance with the guidelines for animal experiments of the College of Bioresource Sciences, Nihon University (Approval No. AP17B066 by Nihon University). Common bottlenose dolphins were treated with care during all experiments and under the supervision of veterinarians. Beagle dogs were reared under the appropriate management at the Institute for Animal Reproduction (Kasumigaura, Ibaraki, Japan), founded with the permission of the Ministry of Education, Culture, Sports, Science and Technology of Japan.

### Experimental design

To characterize plasma metabolites in the common bottlenose dolphin, a combination of CE-TOFMS and LC-TOFMS was applied to detect metabolites. As these analyses are expensive, we sought to characterize plasma metabolites within a modest budget by analyzing samples from only three healthy, young mature female common bottlenose dolphins in captivity. The data obtained were compared with simultaneously-analyzed data from beagle dogs, which we selected as a representative model animal for terrestrial carnivores. The analyses from both species were performed using the same conditions with regard to gender, feeding, and sampling times to remove the influence of these factors^11^.

### Blood sampling

In January 2017, blood samples were collected from the vein of tail flukes by a husbandry method between 9:00–10:00 from three sexually mature female common bottlenose dolphins, *Tursiops truncatus* at the Port of Nagoya Public Aquarium (Nagoya, Aichi, Japan); two dolphins kept since 2005 were ca. 15–18 years old and the third dolphin reared since 2001 was 19 years old. Blood collection was also carried out in three mature female beagle dogs (56, 60, and 62 months old) kept at the Institute for Animal Reproduction. All animals were fed at 16:00 on the day before of sampling and had fasted until blood samples were taken; the animals had no obvious health problems on the basis of blood chemistry and behavior. Usually, the dolphins were fed with chub mackerel, Atka mackerel, and herring that are commonly fed to captive dolphins; and the dogs were given a compound feed diet (CD-5M, CLEA Japan Inc., Tokyo, Japan). Blood samples were placed into EDTA 2Na-contained vacuum blood collection tubes and centrifuged at 3,000 × g for 10 min at room temperature. Plasma was transferred into a cryopreservation tube and stored in liquid nitrogen until analysis.

### Metabolomic analysis

Metabolomic measurements were performed by Human Metabolome Technologies Inc. (HMT; Yamagata, Japan). Low-molecular compounds in the plasma were detected by a “Dual Scan” analysis using CE-TOFMS and LC-TOFMS.

### CE-TOFMS

Fifty μL of plasma were added to 450 μL of methanol with an internal standard (10 μM final concentration solution of methionine sulfone and 10-camphorsulfonic acid, solution ID: H3304-1002, HMT) at 0 °C in order to inactivate enzymes. The extract was further mixed with 500 μL chloroform and 200 μL Milli-Q water. The mixture was centrifuged at 2300 × g for 5 min at 4 °C, and 350 μL of the upper aqueous layer was transferred into an ultrafiltration tube (Ultra-free MC PLHCC, filter-unit 5 kDa, HMT) and filtered to remove proteins. The filtrate was centrifugally concentrated and rehydrated in 50 μL of Milli-Q water for injection into the CE-TOFMS.

CE-TOFMS measurement was carried out using an Agilent CE Capillary Electrophoresis System equipped with an Agilent 6210 time of flight mass spectrometer, Agilent 1100 isocratic HPLC pump, Agilent G1603A CE-MS adapter kit, and Agilent G1607A CE-ESI-MS sprayer kit (Agilent Technologies, Waldbronn, Germany). The systems were controlled by Agilent G2201AA ChemStation software version B.03.01 for CE (Agilent Technologies). Pre-treated samples were applied into the system using fused silica capillaries (50 μm i.d. ×80 cm total length) (Agilent Technologies) to detect hydrosoluble metabolites. The measurement modes for cation and anion metabolites are shown in Table S1.

### LC-TOFMS

Five hundred μL of plasma were mixed with 1500 μL 1% formic acid/acetonitrile with the same internal standard above (Solution ID: H3304-1002, HMT) at 0 °C in order to inactivate enzymes, and the mixture was centrifuged at 2300 × g for 5 min at 4 °C. The supernatant was filtered using a Hybrid SPE Phospholipid 55261-U (Supelco, Bellefonte, PA, USA) to remove phospholipids. The filtrate was desiccated and the residue was dissolved in 100 μL 50% isopropanol (v/v) for injection into the LC-TOFMS.

LC-TOFMS measurement was carried out using an Agilent LC System (Agilent 1200 series RRLC system SL) equipped with an Agilent 6230 Time of Flight mass spectrometer (Agilent Technologies). The systems were controlled by the same software as described above. Pre-treated samples were applied to an Agilent 1200 series RRLC system SL with ODS column (2 × 50 mm, 2 μm) equipped with an Agilent LC/MSD TOF (Agilent Technologies) to analyze lipophilic metabolites. The cationic and anionic compounds were measured using an ODS column (2 × 50 mm, 2 μm) as described previously^58^. The measurement modes for cation and anion metabolites are shown in Table S1.

### Peak analysis

The information on peaks was extracted using the automatic integration software MasterHands ver. 2.17.1.11 (Keio University, Tsuruoka, Yamagata Japan) to obtain mass-to-charge ratio (*m*/*z*), migration time (MT) for CE-TOFMS measurement, or retention time (RT) for LC-TOFMS measurement, and peak area^59^. Signal peaks corresponding to isotopomers, adduct ions, and other product ions of known metabolites were excluded, and remaining peaks were annotated with putative metabolites from the HMT metabolite database based on their MT/RT and m/z values determined by TOFMS. The tolerance range for the peak annotation was configured at ±0.5 min for MT and ±0.3 min for RT, and ±10 and ±20 ppm for m/z for CE and LC-TOFMS. Peak areas were normalized against those of the internal standards and the resultant relative area values were further normalized by sample size.

A principal component analysis (PCA), a hierarchical cluster analysis and heatmap creation were performed using SampleStat software (HMT). To analyze the similarities of plasma metabolites among individuals, a cluster analysis with Ward’s method was conducted using Microsoft Excel on the relative area data of all 250 metabolites that were detected in either species. For this analysis, the value of data for metabolites showing no peaks was substituted with zero. Detected metabolites were plotted on metabolic pathway maps using VANTED (Visualization and Analysis of Networks containing Experimental Data) software^60^.

### Statistical analysis

Welch’s *t* test was applied to test the differences in relative areas of metabolites in the plasma of dolphins and dogs using PeakStat (HMT). All values are presented as means ± standard deviation (S.D.).

## Electronic supplementary material

Supplementary Information
