# Supplementary material for: Plasma metabolomic analysis in mature female common bottlenose dolphins: profiling the characteristics of metabolites after overnight fasting by comparison with data in beagle dogs
Source: Sci Rep. 2018 Aug 13;8:12030. doi: 10.1038/s41598-018-30563-x (PMC6089887; doi:10.1038/s41598-018-30563-x)
Supplement: Supplementary file 1 — Supplementary Information [file 41598_2018_30563_MOESM1_ESM.docx]

**Supplementary Information**

Plasma metabolomic analysis in mature female common bottlenose dolphins: profiling the characteristics of metabolites after overnight fasting by comparison with data in beagle dogs

Miwa Suzuki, Motoi Yoshioka, Yoshito Ohno, Yuichiro Akune

**S1 Table**. Measurement mode for CE-TOFMS and LC-TOFMS.

| **CE-TOFMS** | | |
| --- | --- | --- |
|  | **Cation mode** | **Anion mode** |
| Run buffer | Cation buffer solution  (p/n: H3301-1001) | Anion bugger solution  (p/n: H3302-1021) |
| Rinse buffer | Cation buffer solution  (p/n: H3301-1001) | Anion bugger solution  (p/n: H3302-1021) |
| Sample injection | Pressure injection 50 mbar,  10 sec | Pressure injection 50 mbar,  25 sec |
| CE voltage | Positive, 27 kV | Positive, 30 kV |
| MS ionization mode | ESI Positive | ESI Negative |
| MS capillary voltage | 4,000 V | 3,500 V |
| MS scan range | m/z 50-1,000 | |
| Sheath liquid | HMT Sheath Liquid (p/n: H3301-1020) | |
| **LC-TOFMS** | | |
|  | **Positive mode** | **Negative mode** |
| Column temperature | 40 °C | |
| Mobile phase | A: H_2_O (65:30:5)/0.1% HCOOH | |
|  | B: Isopropanol:acetonitrile:H_2_O(65:30:5)/0.1% HCOOH, 2 mM HCOONH_4_ | |
| Flow rate | 0.3 mL / min | |
| Run time | 20 min | |
| Post time | 7.5 min | |
| Gradient condition | 0-0.5 min: B 1%, 0.5-13.5: B 1-100%, 13.5-20 min: B 100% | |
| MS ionization mode | ESI Positive | ESI Negative |
| MS Nebulizer pressure | 40 psi | |
| MS dry gas glow | 10 L / min | |
| MS dry gas temperature | 350 °C | |
| MS capillary voltage | 3,500 V | |
| MS scan range | m/z 100-1,700 | |
| Sample injection | 1μL | |

**S2 Table**. Relative peak areas of detected metabolites in plasma of common bottlenose dolphins and beagle dogs.

| Compound name | KEGG ID | Bottlenose dolphin | | Beagle dog | | Dolphin vs beagle dog | | |
| --- | --- | --- | --- | --- | --- | --- | --- | --- |
|  |  | Mean | S.D. | Mean | S.D. | Ratio | p-value |  |
| 1-Aminocyclopentanecarboxylic acid | C03969 | 9.7E-04 | 4.0E-04 | N.A. | N.A. | 1< | N.A. |  |
| 1-Methyl-4-imidazoleacetic acid | C05828 | 4.0E-04 | 2.9E-04 | N.A. | N.A. | 1< | N.A. |  |
| 1-Methylhistidine 3-Methylhistidine | No ID | 1.4E-01 | 2.5E-02 | 1.8E-03 | 4.9E-04 | 78 | 0.010 | * |
| 1-Methylnicotinamide | C01152 | 3.7E-05 | 1.3E-05 | 2.6E-04 | 8.1E-05 | 0.14 | 0.039 | * |
| 11-Aminoundecanoic acid | C02918 | 1.5E-04 | 4.2E-05 | 1.4E-04 | 3.0E-05 | 1.0 | 0.917 |  |
| 15(S)-HETE | C19325 | N.A. | N.A. | 1.7E-06 | 5.2E-07 | <1 | N.A. |  |
| 1*H*-Imidazole-4-propionic acid | C04742 | N.A. | N.A. | 1.8E-04 | 4.5E-05 | <1 | N.A. |  |
| 2'-Deoxycytidine | No ID | 6.3E-05 | 7.5E-06 | 2.5E-04 | 4.1E-05 | 0.3 | 0.014 | * |
| 2-Aminoisobutyric acid 2-Aminobutyric acid | C00881 | 1.4E-02 | 6.3E-03 | 4.6E-03 | 6.9E-04 | 2.9 | 0.131 |  |
| 2-Hydroxy-4-methylvaleric acid | C03665 | 6.1E-04 | 6.3E-04 | N.A. | N.A. | 1< | N.A. |  |
| 2-Hydroxybutyric acid | C02261 | 2.9E-03 | 1.1E-03 | 1.0E-03 | 1.3E-04 | 2.9 | 0.088 |  |
| 2-Hydroxyisobutyric acid | C03264 | 1.4E-03 | 9.2E-04 | 1.5E-04 | 3.7E-05 | 9.4 | 0.144 |  |
| 2-Hydroxytetradecanoic acid | C05984 | 9.6E-07 | 7.8E-08 | 9.3E-07 | 4.7E-08 | 1.0 | 0.631 |  |
| 2-Hydroxyvaleric acid | No ID | 8.1E-04 | 8.4E-05 | 1.8E-04 | 8.1E-05 | 4.4 | 7.4E-04 | *** |
| 2-Oxoglutaric acid | C13790 | N.A. | N.A. | 1.2E-03 | 2.7E-04 | <1 | N.A. |  |
| 2-Oxoisovaleric acid | No ID | 1.1E-03 | 2.1E-04 | 8.2E-04 | 7.5E-05 | 1.4 | 0.113 |  |
| 2-Quinolinecarboxylic acid | C00026 | N.A. | N.A. | 3.7E-04 | 2.3E-04 | <1 | N.A. |  |
| 3-Aminobutyric acid | C00141 | 8.5E-05 | 8.6E-06 | 1.5E-04 | 1.1E-05 | 0.6 | 0.001 | ** |
| 3-Hydroxybutyric acid | C06325 | 1.1E-03 | 2.4E-04 | 1.2E-03 | 8.5E-05 | 0.9 | 0.627 |  |
| 3-Hydroxytetradecanoic acid | No ID | 2.6E-06 | 1.2E-06 | N.A. | N.A. | 1< | N.A. |  |
| 3-Indoxylsulfuric acid | C01089 | 4.1E-04 | 2.2E-04 | 1.9E-03 | 4.9E-04 | 0.2 | 0.019 | * |
| 3-Methoxytyrosine | No ID | 3.2E-04 | 1.9E-05 | 1.1E-04 | 2.7E-05 | 2.8 | 7.6E-04 | *** |
| 4-Aminohippuric acid | No ID | 4.0E-05 | 1.4E-05 | N.A. | N.A. | 1< | N.A. |  |
| 4-Methyl-2-oxovaleric acid 3-Methyl-2-oxovaleric acid | No ID | 7.7E-03 | 9.3E-04 | 5.0E-03 | 1.1E-04 | 1.6 | 0.034 | * |
| 4-Pyridoxic acid | No ID | 1.0E-04 | 1.9E-05 | N.A. | N.A. | 1< | N.A. |  |
| 5-Amino-4-oxovaleric acid | C00233 | 3.6E-05 | 1.2E-05 | 3.7E-05 | 3.2E-06 | 1.0 | 0.942 |  |
| 5-Aminovaleric acid | C00671 | N.A. | N.A. | 6.7E-04 | N.A. | <1 | N.A. |  |
| 5-Hydroxylysine | C00847 | 7.7E-05 | 2.9E-05 | 4.3E-05 | 6.0E-07 | 1.8 | 0.180 |  |
| 5-Methoxyindoleacetic acid | C00430 | 6.4E-04 | 4.9E-05 | 5.3E-04 | 2.2E-05 | 1.2 | 0.048 | * |
| 5-Oxo-2-tetrahydrofurancarboxylic acid | C00431 | N.A. | N.A. | 2.6E-04 | 1.5E-05 | <1 | N.A. |  |
| 5-Oxoproline | C16741 | 6.7E-04 | 1.7E-04 | 9.2E-04 | 7.5E-05 | 0.7 | 0.108 |  |
| 7-Methylguanine | C05660 | 4.4E-05 | 1.1E-05 | 5.8E-05 | 1.4E-05 | 0.8 | 0.238 |  |
| AC(12:0) | No ID | 1.3E-05 | 3.7E-06 | N.A. | N.A. | 1< | N.A. |  |
| AC(12:1)-1 | C01879 | 1.2E-06 | 5.7E-07 | 1.1E-06 | 1.3E-07 | 1.1 | 0.788 |  |
| AC(12:1)-2 | C02242 | 7.5E-06 | 4.0E-06 | 7.7E-06 | 3.4E-06 | 1.0 | 0.936 |  |
| AC(12:1)-3 | No ID | 8.3E-06 | 3.7E-06 | 4.0E-06 | 1.1E-06 | 2.1 | 0.169 |  |
| AC(13:1)-1 | No ID | 1.9E-06 | N.A. | N.A. | N.A. | 1< | N.A. |  |
| AC(13:1)-2 | No ID | 1.3E-06 | 2.4E-07 | 9.6E-07 | 5.6E-08 | 1.4 | 0.106 |  |
| AC(14:1)-1 | No ID | 1.9E-06 | N.A. | N.A. | N.A. | 1< | N.A. |  |
| AC(14:1)-2 | No ID | 3.0E-06 | 1.0E-07 | N.A. | N.A. | 1< | N.A. |  |
| AC(14:1)-3 | No ID | 9.7E-06 | 5.7E-06 | 2.6E-06 | 1.4E-06 | 3.7 | 0.159 |  |
| AC(14:2) | No ID | 1.6E-06 | 3.1E-07 | 8.2E-06 | 4.7E-06 | 0.2 | 0.138 |  |
| AC(14:3) | No ID | 9.9E-06 | 4.8E-06 | 9.7E-07 | N.A. | 10 | N.A. |  |
| AC(15:0) | No ID | 1.0E-05 | 2.4E-06 | 9.4E-06 | 2.2E-06 | 1.1 | 0.669 |  |
| AC(16:1) | No ID | 4.1E-06 | 9.1E-07 | N.A. | N.A. | 1< | N.A. |  |
| AC(17:0) | No ID | 4.0E-06 | 4.2E-07 | 3.7E-06 | 1.4E-06 | 1.1 | 0.761 |  |
| AC(18:0) | No ID | 2.5E-06 | N.A. | 1.1E-06 | 1.2E-07 | 2.2 | N.A. |  |
| AC(20:0) | No ID | 4.6E-06 | 9.2E-07 | 1.1E-06 | 1.3E-07 | 4.3 | 0.019 | * |
| AC(20:1) | No ID | 4.4E-06 | 3.3E-06 | N.A. | N.A. | 1< | N.A. |  |
| AC(21:0) | No ID | 9.2E-07 | N.A. | N.A. | N.A. | 1< | N.A. |  |
| AC(22:0) | No ID | 1.6E-06 | 2.9E-07 | N.A. | N.A. | 1< | N.A. |  |
| Acetoacetic acid | No ID | 2.3E-04 | N.A. | N.A. | N.A. | 1< | N.A. |  |
| ADMA | No ID | 2.6E-04 | 8.1E-05 | 4.8E-04 | 1.1E-04 | 0.6 | 0.053 |  |
| Ala | No ID | 6.2E-02 | 3.2E-03 | 6.1E-02 | 3.7E-03 | 1.0 | 0.602 |  |
| Anserine_divalent | C00164 | 6.7E-03 | 2.0E-03 | 9.9E-04 | 2.7E-04 | 6.8 | 0.037 | * |
| Arachidic acid | C03626 | 2.2E-06 | 1.4E-06 | 1.3E-06 | 2.8E-07 | 1.8 | 0.359 |  |
| Arachidonic acid | C00041 | 2.6E-04 | 2.3E-04 | 2.6E-04 | 6.6E-05 | 1.0 | 0.998 |  |
| Arg | C01262 | 2.9E-02 | 1.3E-03 | 2.7E-02 | 5.5E-03 | 1.1 | 0.710 |  |
| Argininosuccinic acid | C06425 | 2.4E-05 | N.A. | 1.6E-05 | N.A. | 1.5 | N.A. |  |
| Asiatic acid | C00219 | N.A. | N.A. | 2.4E-06 | N.A. | <1 | N.A. |  |
| Asn | C00062 | 7.4E-03 | 2.0E-03 | 7.6E-03 | 1.0E-03 | 1.0 | 0.874 |  |
| Asp | C03406 | 4.1E-04 | 7.3E-06 | 8.0E-04 | 1.8E-04 | 0.5 | 0.064 |  |
| Astaxanthin | C08617 | 2.1E-06 | 1.4E-06 | N.A. | N.A. | 1< | N.A. |  |
| Azetidine 2-carboxylic acid | C00152 | 1.8E-04 | 1.2E-04 | N.A. | N.A. | 1< | N.A. |  |
| Behenic acid | C00049 | 1.7E-06 | N.A. | 8.4E-07 | 2.5E-07 | 2.1 | N.A. |  |
| Betaine | C08580 | 1.3E-02 | 1.1E-03 | 7.5E-02 | 4.6E-03 | 0.2 | 0.001 | ** |
| Campesterol | C08267 | N.A. | N.A. | 4.8E-06 | 1.4E-06 | <1 | N.A. |  |
| Carnitine | C08281 | 7.0E-03 | 1.5E-03 | 5.3E-03 | 1.2E-03 | 1.3 | 0.204 |  |
| Carnosine | C00719 | 5.6E-04 | 2.6E-04 | 1.9E-03 | 2.6E-04 | 0.3 | 0.003 | ** |
| Carvone | C01789 | 1.1E-05 | 4.4E-07 | 1.2E-05 | 6.5E-07 | 1.0 | 0.340 |  |
| Chenodeoxycholic acid | C00318 | N.A. | N.A. | 2.5E-06 | 2.0E-06 | <1 | N.A. |  |
| Cholesterol | C00386 | 6.1E-03 | 1.3E-03 | 5.0E-03 | 7.8E-04 | 1.2 | 0.279 |  |
| Cholesterol sulfate | C01767 | 3.7E-04 | 1.0E-04 | 1.6E-05 | 3.8E-06 | 24 | 0.026 | * |
| Cholic acid | C02528 | 8.2E-07 | 2.0E-08 | 1.2E-04 | 1.3E-04 | 0.007 | 0.245 |  |
| Choline | C00187 | 4.6E-03 | 1.6E-04 | 2.7E-03 | 9.7E-04 | 1.7 | 0.068 |  |
| cis-11,14-Eicosadienoic acid-1 | C18043 | 1.5E-06 | N.A. | N.A. | N.A. | 1< | N.A. |  |
| cis-11,14-Eicosadienoic acid-2 | C00695 | 2.1E-05 | 1.6E-05 | 1.1E-05 | 2.3E-06 | 1.9 | 0.383 |  |
| *cis*-11-Eicosenoic acid | C00114 | 8.1E-04 | 6.9E-04 | 6.0E-05 | 1.8E-05 | 14 | 0.199 |  |
| *cis*-13-Eicosenoic acid | No ID | 9.1E-07 | 1.0E-07 | N.A. | N.A. | 1< | N.A. |  |
| *cis*-4,7,10,13,16,19-Docosahexaenoic acid | No ID | 7.9E-04 | 6.8E-04 | 3.5E-05 | 1.4E-05 | 23 | 0.193 |  |
| *cis*-5,8,11,14,17-Eicosapentaenoic acid Abietic acid | C16526 | 3.2E-03 | 3.3E-03 | 3.3E-05 | 1.1E-05 | 97 | 0.243 |  |
| *cis*-8,11,14-Eicosatrienoic acid | No ID | 1.9E-05 | 1.6E-05 | 3.0E-05 | 9.7E-06 | 0.6 | 0.391 |  |
| *cis*-Aconitic acid | C06429 | 9.4E-04 | 2.7E-04 | 9.2E-04 | 1.1E-04 | 1.0 | 0.910 |  |
| Citric acid | C06428 | 3.0E-02 | 1.1E-02 | 2.1E-02 | 1.8E-03 | 1.5 | 0.281 |  |
| Citrulline | C06087 | 5.0E-03 | 2.6E-03 | 9.1E-03 | 2.2E-03 | 0.6 | 0.113 |  |
| Corticosterone Cortexolone | C03242 | 2.8E-06 | 1.8E-06 | 9.2E-07 | N.A. | 3.0 | N.A. |  |
| Cortisol | C00417 | 8.9E-06 | 4.3E-06 | 3.8E-06 | 2.2E-06 | 2.4 | 0.164 |  |
| Cortisone | C00158 | 1.6E-06 | 1.5E-07 | 2.6E-06 | 7.4E-07 | 0.6 | 0.139 |  |
| Creatine | C00327 | 3.4E-02 | 1.3E-03 | 6.9E-03 | 2.1E-03 | 5.0 | 1.8E-04 | *** |
| Creatinine | C02140 | 3.1E-02 | 9.2E-03 | 9.0E-03 | 1.0E-03 | 3.4 | 0.052 |  |
| Cystathionine | C05488 | 2.0E-04 | 4.0E-05 | 5.6E-04 | 1.5E-04 | 0.3 | 0.045 | * |
| Cysteine glutathione disulfide | C00735 | 3.6E-05 | 1.1E-05 | 4.9E-04 | 3.4E-05 | 0.07 | 7.9E-04 | *** |
| Cystine | C00762 | 6.5E-03 | 2.6E-04 | 5.4E-03 | 4.0E-04 | 1.2 | 0.021 | * |
| Cytidine | C00300 | 9.8E-05 | 4.7E-05 | 1.9E-04 | 4.5E-05 | 0.5 | 0.075 |  |
| Cytosine | C00791 | N.A. | N.A. | 2.9E-05 | N.A. | <1 | N.A. |  |
| Daidzein | C00542 | N.A. | N.A. | 6.4E-07 | N.A. | <1 | N.A. |  |
| Decanoic acid | C05526 | 2.4E-04 | 4.5E-05 | 1.3E-04 | N.A. | 1.8 | N.A. |  |
| Deoxycholic acid | C00491 | N.A. | N.A. | 1.5E-05 | 1.5E-05 | <1 | N.A. |  |
| Desmosterol | C00475 | 1.9E-06 | 4.2E-07 | N.A. | N.A. | 1< | N.A. |  |
| Diethanolamine | C00380 | 2.4E-04 | 4.1E-05 | 3.7E-04 | 1.5E-04 | 0.6 | 0.259 |  |
| Dyphylline | C10208 | 4.4E-02 | 3.1E-03 | 3.9E-02 | 1.9E-03 | 1.1 | 0.094 |  |
| Ectoine | C01571 | 3.0E-04 | 1.1E-04 | 9.4E-05 | N.A. | 3.2 | N.A. |  |
| Erucic acid | C04483 | 5.2E-04 | 4.5E-04 | 9.7E-05 | 1.9E-05 | 5.4 | 0.247 |  |
| Ethanolamine | C01802 | 4.7E-04 | 8.9E-05 | 6.8E-04 | 9.5E-05 | 0.7 | 0.052 |  |
| Ethanolamine phosphate | C06772 | 9.6E-05 | 3.3E-05 | 2.1E-04 | 6.3E-05 | 0.5 | 0.069 |  |
| FA(12:0) | C07819 | 5.0E-06 | 3.8E-06 | 1.4E-06 | 3.7E-07 | 3.7 | 0.246 |  |
| FA(13:0) | C06231 | 4.9E-05 | 2.4E-05 | N.A. | N.A. | 1< | N.A. |  |
| FA(14:1)-1 FA(14:1)-2-2 | C08316 | 1.7E-05 | 1.7E-05 | 3.9E-06 | 1.5E-06 | 4.4 | 0.306 |  |
| FA(14:1)-2-1 | C00189 | 7.6E-06 | 6.6E-06 | N.A. | N.A. | 1< | N.A. |  |
| FA(14:2) | C00346 | 1.3E-06 | N.A. | N.A. | N.A. | 1< | N.A. |  |
| FA(14:3) | No ID | 6.2E-06 | 1.9E-06 | 5.4E-06 | 9.6E-07 | 1.2 | 0.522 |  |
| FA(15:0)-1 | No ID | 1.2E-06 | 4.2E-07 | N.A. | N.A. | 1< | N.A. |  |
| FA(15:0)-2 | No ID | 1.3E-05 | 8.0E-06 | 1.8E-06 | 5.8E-07 | 7.5 | 0.130 |  |
| FA(15:1)-1-1 FA(15:1)-2-1 | No ID | 4.2E-06 | 2.9E-06 | N.A. | N.A. | 1< | N.A. |  |
| FA(15:1)-1-2 FA(15:1)-2-2 | No ID | 8.6E-06 | 5.2E-06 | N.A. | N.A. | 1< | N.A. |  |
| FA(16:2) | No ID | 9.1E-05 | 9.4E-05 | 1.1E-06 | 1.2E-07 | 81 | 0.239 |  |
| FA(16:3)-1 | No ID | 2.1E-06 | 1.7E-06 | 7.8E-07 | N.A. | 2.7 | N.A. |  |
| FA(16:3)-2 | No ID | 1.8E-05 | 1.6E-05 | N.A. | N.A. | 1< | N.A. |  |
| FA(17:0)-1 | No ID | 1.9E-06 | 8.8E-07 | N.A. | N.A. | 1< | N.A. |  |
| FA(17:0)-2 Heptadecanoic acid-1 | No ID | 1.9E-05 | 8.4E-06 | 1.9E-05 | 5.7E-06 | 1.0 | 0.918 |  |
| FA(17:1) | No ID | 1.1E-04 | 9.4E-05 | 2.4E-05 | 5.4E-06 | 4.6 | 0.258 |  |
| FA(17:2) | No ID | 9.1E-06 | 7.2E-06 | 8.2E-07 | N.A. | 11 | N.A. |  |
| FA(19:0)-1 | No ID | 2.2E-06 | N.A. | 6.1E-07 | N.A. | 3.6 | N.A. |  |
| FA(19:0)-2 | No ID | 1.1E-06 | 5.6E-07 | 1.3E-06 | 4.1E-07 | 0.8 | 0.678 |  |
| FA(19:1) | No ID | 1.8E-05 | 1.1E-05 | 7.7E-06 | 2.0E-06 | 2.3 | 0.258 |  |
| FA(19:2) | No ID | 4.4E-06 | 4.2E-06 | N.A. | N.A. | 1< | N.A. |  |
| FA(20:3) | No ID | 4.1E-06 | 4.3E-06 | 7.4E-06 | 2.5E-07 | 0.5 | 0.304 |  |
| FA(22:2)-1 | No ID | 1.6E-06 | 6.6E-07 | N.A. | N.A. | 1< | N.A. |  |
| FA(22:2)-2 | No ID | 3.2E-06 | 2.2E-06 | 1.1E-06 | 2.7E-07 | 3.0 | 0.234 |  |
| FA(22:4) | No ID | 3.0E-05 | 2.9E-05 | 2.3E-05 | 4.8E-06 | 1.3 | 0.714 |  |
| FA(22:5)-1 | No ID | 7.1E-05 | 6.2E-05 | 4.1E-06 | 1.5E-06 | 17 | 0.202 |  |
| FA(22:5)-2 | No ID | 1.8E-04 | 1.4E-04 | 3.7E-05 | 1.1E-05 | 4.9 | 0.230 |  |
| FA(24:2) | No ID | 3.0E-06 | N.A. | N.A. | N.A. | 1< | N.A. |  |
| FA(24:4) | No ID | 4.0E-06 | 2.4E-06 | 1.1E-06 | 1.0E-07 | 3.5 | 0.177 |  |
| FA(24:5) | No ID | 9.1E-06 | 6.6E-06 | N.A. | N.A. | 1< | N.A. |  |
| FA(25:3) | No ID | N.A. | N.A. | 3.4E-06 | 9.1E-07 | <1 | N.A. |  |
| FA(26:2) | No ID | 7.2E-06 | 4.4E-06 | N.A. | N.A. | 1< | N.A. |  |
| GABA | No ID | 9.0E-05 | N.A. | N.A. | N.A. | 1< | N.A. |  |
| Genistein Baicalein | No ID | N.A. | N.A. | 5.7E-07 | N.A. | <1 | N.A. |  |
| Gln | No ID | 9.5E-02 | 1.2E-02 | 1.4E-01 | 9.2E-04 | 0.7 | 0.024 | * |
| Glu | No ID | 7.4E-03 | 8.7E-04 | 7.4E-03 | 9.4E-04 | 1.0 | 0.953 |  |
| Gluconic acid | No ID | N.A. | N.A. | 3.9E-04 | 7.9E-05 | <1 | N.A. |  |
| Glucuronic acid-1 Galacturonic acid-1 | No ID | N.A. | N.A. | 1.3E-04 | 7.7E-06 | <1 | N.A. |  |
| Glucuronic acid-2 Galacturonic acid-2 | No ID | N.A. | N.A. | 1.2E-04 | 3.1E-05 | <1 | N.A. |  |
| Glutathione (GSSG)_divalent | No ID | N.A. | N.A. | 2.0E-04 | 1.4E-05 | <1 | N.A. |  |
| Gly | No ID | 2.8E-02 | 5.6E-03 | 1.7E-02 | 2.1E-03 | 1.6 | 0.068 |  |
| Gly-Gly | C00334 | 4.3E-05 | 4.5E-06 | 5.6E-05 | 7.2E-06 | 0.8 | 0.088 |  |
| Glycerol | C06563 | 1.8E-01 | 3.5E-02 | 1.8E-01 | 2.7E-02 | 1.0 | 0.960 |  |
| Glycerol 3-phosphate | C10023 | N.A. | N.A. | 1.6E-04 | 3.1E-05 | <1 | N.A. |  |
| Glycerophosphocholine | C00064 | 1.1E-04 | 6.7E-05 | 2.4E-04 | 7.5E-05 | 0.5 | 0.155 |  |
| Glycitein | C00025 | N.A. | N.A. | 1.3E-06 | 3.4E-07 | <1 | N.A. |  |
| Glycocholic acid | C00257 | 9.3E-07 | 4.2E-07 | N.A. | N.A. | 1< | N.A. |  |
| Glyoxylic acid | No ID | 1.8E-04 | N.A. | 1.7E-04 | N.A. | 1.1 | N.A. |  |
| Guanidinosuccinic acid | No ID | 2.1E-04 | 4.6E-05 | N.A. | N.A. | 1< | N.A. |  |
| Guanidoacetic acid | No ID | 2.8E-04 | 7.6E-05 | 5.4E-04 | 1.4E-04 | 0.5 | 0.057 |  |
| Guanine | No ID | 1.5E-04 | 3.3E-05 | N.A. | N.A. | 1< | N.A. |  |
| Hecogenin | C00127 | 7.7E-05 | 1.3E-05 | 1.3E-05 | 1.5E-06 | 6.0 | 0.012 | * |
| Heptadecanoic acid-2 | C00037 | 2.5E-05 | 1.6E-05 | 1.5E-05 | 3.9E-06 | 1.7 | 0.379 |  |
| Hippuric acid | C02037 | 1.9E-04 | 3.5E-05 | N.A. | N.A. | 1< | N.A. |  |
| His | C00116 | 1.6E-02 | 1.8E-03 | 2.3E-02 | 1.1E-03 | 0.7 | 0.009 | ** |
| Homocarnosine | C00093 | 1.8E-03 | 7.6E-04 | 2.0E-04 | 5.1E-05 | 8.9 | 0.068 |  |
| Homocitrulline | C00670 | 2.9E-03 | 1.7E-03 | 2.7E-04 | 5.3E-05 | 11 | 0.113 |  |
| Homocystine | C14536 | 1.1E-04 | 1.7E-05 | N.A. | N.A. | 1< | N.A. |  |
| Homoserine | C01921 | 5.8E-05 | 2.7E-05 | 4.5E-05 | 5.8E-06 | 1.3 | 0.494 |  |
| Homovanillic acid | C00048 | 1.7E-04 | 2.9E-05 | 2.6E-04 | 9.7E-05 | 0.7 | 0.244 |  |
| Hydroxyproline | C03139 | 4.7E-03 | 1.5E-03 | 1.5E-03 | 1.2E-04 | 3.1 | 0.069 |  |
| Hypotaurine | C00581 | 1.0E-04 | 4.1E-05 | 5.8E-05 | 7.0E-06 | 1.8 | 0.193 |  |
| Hypoxanthine | C00242 | 1.3E-03 | 1.2E-04 | 5.3E-05 | 5.0E-06 | 25 | 0.003 | ** |
| Ile | C08902 | 2.8E-02 | 3.5E-03 | 1.9E-02 | 7.7E-04 | 1.4 | 0.047 | * |
| Imidazole-4-acetic acid | No ID | 6.9E-05 | 4.3E-05 | 7.8E-05 | 1.6E-05 | 0.9 | 0.805 |  |
| Imidazolelactic acid | C01586 | 6.2E-05 | 5.7E-06 | 5.8E-05 | 3.4E-06 | 1.1 | 0.483 |  |
| Indole-3-carboxaldehyde | C00135 | N.A. | N.A. | 6.4E-06 | 8.4E-07 | <1 | N.A. |  |
| Inosine | C00884 | 7.1E-05 | 7.6E-06 | N.A. | N.A. | 1< | N.A. |  |
| Isethionic acid | C02427 | 8.5E-04 | 1.1E-04 | 6.9E-04 | 3.8E-05 | 1.2 | 0.101 |  |
| Isocitric acid | C01817 | 7.6E-04 | 2.4E-04 | 6.6E-04 | 1.0E-04 | 1.2 | 0.529 |  |
| Isofraxidin | C00263 | N.A. | N.A. | 1.1E-06 | N.A. | <1 | N.A. |  |
| Isovaleric acid Valeric acid | C05582 | 3.6E-04 | N.A. | N.A. | N.A. | 1< | N.A. |  |
| Isovalerylalanine *N*-Acetylleucine | C01157 | N.A. | N.A. | 2.1E-04 | 7.5E-05 | <1 | N.A. |  |
| Kynurenic acid | C00519 | N.A. | N.A. | 2.2E-05 | 1.8E-05 | <1 | N.A. |  |
| Kynurenine | C00262 | 8.6E-04 | 3.2E-05 | 1.1E-03 | 2.1E-04 | 0.8 | 0.217 |  |
| Lactic acid | C00407 | 3.0E-02 | 2.1E-02 | 7.6E-02 | 1.6E-02 | 0.4 | 0.046 | * |
| Lauric acid | C02835 | 1.8E-03 | 7.2E-05 | 1.7E-03 | 1.7E-04 | 1.1 | 0.258 |  |
| Leu | C05568 | 6.0E-02 | 8.2E-03 | 4.2E-02 | 3.5E-03 | 1.4 | 0.047 | * |
| Linoleic acid | C08493 | 4.6E-04 | 4.0E-04 | 1.9E-03 | 3.8E-04 | 0.2 | 0.010 | ** |
| Linolenic acid | C00294 | 2.7E-04 | 2.5E-04 | 8.1E-05 | 2.7E-05 | 3.3 | 0.322 |  |
| Liquiritigenin | C05123 | N.A. | N.A. | 3.6E-06 | N.A. | <1 | N.A. |  |
| Lithocholic acid | C00311 | N.A. | N.A. | 8.8E-07 | N.A. | <1 | N.A. |  |
| Lys | C17480 | 2.0E-02 | 2.3E-03 | 2.8E-02 | 1.5E-03 | 0.7 | 0.015 | * |
| Malic acid | C08262 | 3.1E-04 | 5.7E-05 | 7.5E-04 | 1.2E-04 | 0.4 | 0.011 | * |
| Met | C00803 | 7.3E-03 | 5.6E-04 | 1.3E-02 | 1.9E-03 | 0.5 | 0.025 | * |
| Methionine sulfoxide | No ID | 9.5E-04 | 8.1E-05 | 9.7E-04 | 2.3E-04 | 1.0 | 0.900 |  |
| Mucic acid | C02710 | 1.3E-04 | 1.3E-05 | 1.9E-04 | 5.2E-05 | 0.7 | 0.344 |  |
| Myristic acid | C01717 | 4.3E-04 | 3.2E-04 | 2.6E-05 | 5.0E-06 | 16 | 0.163 |  |
| Myristoleic acid | C00328 | 2.9E-04 | 2.2E-04 | 1.1E-04 | 2.8E-05 | 2.5 | 0.312 |  |
| *N*,*N*-Dimethylglycine | C00186 | 3.2E-04 | 6.9E-05 | 2.0E-03 | 3.8E-04 | 0.2 | 0.015 | * |
| *N*-Acetyl-β-alanine | C02679 | 3.9E-04 | 9.4E-05 | N.A. | N.A. | 1< | N.A. |  |
| *N*-Acetylgalactosamine *N*-Acetylmannosamine *N*-Acetylglucosamine | C00123 | 2.6E-04 | 7.1E-05 | 1.1E-04 | N.A. | 2.3 | N.A. |  |
| *N*-Acetylglutamine | C01595 | N.A. | N.A. | 1.6E-04 | 2.6E-05 | <1 | N.A. |  |
| *N*-Acetylhistidine | C06427 | N.A. | N.A. | 6.5E-05 | 1.5E-05 | <1 | N.A. |  |
| *N*-Acetyltyrosine ethyl ester | C09762 | 1.1E-06 | N.A. | N.A. | N.A. | 1< | N.A. |  |
| *N*-Formylanthranilic acid | C03990 | 9.3E-07 | 9.3E-08 | N.A. | N.A. | 1< | N.A. |  |
| *N*^1^-Methyl-4-pyridone-5-carboxamide | C00047 | 1.4E-04 | 3.8E-05 | N.A. | N.A. | 1< | N.A. |  |
| *N*^5^-Ethylglutamine | C00149 | 1.2E-03 | 2.0E-04 | 6.2E-03 | 1.1E-03 | 0.2 | 0.013 | * |
| *N*^6^-Acetyllysine | C00073, | 2.9E-04 | 4.8E-05 | 1.3E-04 | N.A. | 2.3 | N.A. |  |
| *N*^6^-Methyllysine | C02989 | 1.6E-03 | 8.8E-04 | 5.6E-04 | 2.1E-05 | 2.8 | 0.180 |  |
| Naringenin | C00879, | N.A. | N.A. | 1.6E-06 | N.A. | <1 | N.A. |  |
| Nervonic acid | C06424 | 6.7E-05 | 4.7E-05 | 6.0E-06 | 5.3E-07 | 11 | 0.154 |  |
| Nicotinamide | C08322 | 1.6E-04 | 1.2E-04 | 1.6E-04 | 4.3E-05 | 1.0 | 0.997 |  |
| *N*_ω-_Methylarginine | C01026 | 3.7E-05 | N.A. | 5.2E-05 | 7.8E-06 | 0.7 | N.A. |  |
| *O*-Acetylcarnitine | C01073 | 3.0E-03 | 1.5E-03 | 1.0E-03 | 2.1E-04 | 2.9 | 0.148 |  |
| *O*-Acetylhomoserine 2-Aminoadipic acid | C01132 | 3.3E-04 | 7.1E-05 | 2.6E-04 | 1.5E-04 | 1.3 | 0.539 |  |
| Oleic acid | C00645 | 9.6E-04 | 8.5E-04 | 4.9E-04 | 9.8E-05 | 1.9 | 0.444 |  |
| Oleoyl ethanolamide-1 | C00140 | 3.5E-06 | 1.0E-07 | 3.4E-06 | 4.9E-07 | 1.0 | 0.979 |  |
| Oleoyl ethanolamide-2 | No ID | 7.4E-05 | 2.5E-05 | 1.5E-05 | 2.0E-06 | 4.9 | 0.055 |  |
| Ornithine | C02997 | 3.4E-03 | 4.5E-04 | 2.7E-03 | 2.6E-04 | 1.3 | 0.093 |  |
| *p*-Hydroxymandelic acid | C01657 | N.A. | N.A. | 1.2E-04 | 1.9E-05 | <1 | N.A. |  |
| Palmitic acid | No ID | 2.0E-03 | 1.2E-03 | 7.7E-04 | 1.4E-04 | 2.5 | 0.231 |  |
| Palmitoleic acid | C05843 | 1.5E-03 | 1.1E-03 | 3.2E-04 | 9.9E-05 | 4.6 | 0.216 |  |
| Palmitoylcarnitine | C01047 | 2.5E-04 | 4.3E-05 | 7.8E-05 | 1.7E-05 | 3.3 | 0.011 | * |
| Palmitoylethanolamide | C02727 | 1.6E-05 | 4.3E-06 | 8.7E-06 | 1.7E-06 | 1.8 | 0.094 |  |
| Pantothenic acid | C02728 | 2.2E-04 | 6.3E-05 | N.A. | N.A. | 1< | N.A. |  |
| Pelargonic acid | C00509 | 2.1E-04 | 9.8E-06 | N.A. | N.A. | 1< | N.A. |  |
| Penicillamine | C08323 | 1.0E-04 | 1.5E-05 | 2.1E-04 | 2.4E-05 | 0.5 | 0.006 | ** |
| Pentadecanoic acid | C00153 | 2.2E-05 | 1.3E-05 | 2.5E-06 | 5.6E-07 | 9.0 | 0.116 |  |
| Phe | No ID | 3.6E-02 | 4.2E-03 | 2.1E-02 | 1.7E-03 | 1.7 | 0.016 | * |
| Phenaceturic acid | C02571 | N.A. | N.A. | 1.2E-04 | 2.4E-05 | <1 | N.A. |  |
| Phosphocreatine | C01077 | 7.9E-05 | N.A. | 1.2E-04 | N.A. | 0.7 | N.A. |  |
| Phosphorylcholine | C00956 | 3.5E-04 | 6.7E-05 | 2.3E-04 | 4.2E-05 | 1.5 | 0.074 |  |
| Phytosphingosine | C00712 | 5.8E-06 | 5.8E-06 | 1.9E-06 | 6.4E-07 | 3.1 | 0.365 |  |
| Picolinic acid | No ID | 1.9E-04 | 7.3E-05 | N.A. | N.A. | 1< | N.A. |  |
| Pipecolic acid | No ID | 6.6E-04 | 2.1E-04 | 1.3E-04 | 7.1E-06 | 5.2 | 0.047 | * |
| Piperidine | C00077 | 2.6E-05 | 3.2E-06 | 2.4E-04 | 3.6E-04 | 0.11 | 0.402 |  |
| Pro | C03198, | 3.5E-02 | 2.5E-03 | 3.2E-02 | 4.1E-03 | 1.1 | 0.451 |  |
| Pyridoxal | C00249 | 3.2E-05 | 4.2E-06 | 3.6E-05 | 1.0E-05 | 0.9 | 0.525 |  |
| Pyrophosphate | C08362 | 7.2E-04 | N.A. | N.A. | N.A. | 1< | N.A. |  |
| Pyruvic acid | C02990 | 2.3E-03 | 2.1E-03 | 3.0E-03 | 2.2E-04 | 0.8 | 0.594 |  |
| Retinol-1 | No ID | N.A. | N.A. | 1.3E-05 | 1.9E-06 | <1 | N.A. |  |
| Retinol-2 | C00864 | 4.3E-06 | 2.0E-06 | 6.7E-05 | 2.8E-06 | 0.06 | 1.6E-05 | *** |
| Ricinoleic acid | C01601 | 5.1E-06 | 4.2E-06 | 6.9E-05 | 1.5E-05 | 0.07 | 0.012 | * |
| *S*-Methylcysteine | C07418 | 1.4E-04 | 1.9E-05 | 4.9E-04 | 7.1E-05 | 0.3 | 0.009 | ** |
| *S*-Methylmethionine | No ID | N.A. | N.A. | 5.1E-05 | 4.2E-06 | <1 | N.A. |  |
| Sarcosine | C00079 | 8.1E-04 | 2.9E-05 | 1.6E-03 | 5.5E-04 | 0.5 | 0.126 |  |
| SDMA | C05598 | 2.9E-04 | 9.4E-05 | 1.2E-04 | 2.8E-05 | 2.4 | 0.077 |  |
| Ser | C02305 | 1.3E-02 | 1.9E-03 | 1.8E-02 | 4.6E-03 | 0.7 | 0.176 |  |
| Sitosterol | C00588 | N.A. | N.A. | 2.8E-06 | 9.0E-07 | <1 | N.A. |  |
| Sphinganine | C12144 | 2.6E-06 | 5.1E-07 | 2.9E-06 | 8.6E-07 | 0.9 | 0.646 |  |
| Sphingosine | C10164 | 5.4E-06 | 1.9E-06 | 4.4E-06 | 1.4E-06 | 1.2 | 0.510 |  |
| Sphingosine 1-phosphate | C00408 | 2.3E-06 | 1.5E-06 | N.A. | N.A. | 1< | N.A. |  |
| Stachydrine | C01746 | 2.1E-04 | 1.0E-04 | 4.0E-04 | 3.7E-05 | 0.5 | 0.077 |  |
| Stearic acid | C00148 | 8.4E-04 | 3.9E-04 | 9.6E-04 | 3.0E-04 | 0.9 | 0.714 |  |
| Stearidonic acid | C00250 | 7.8E-04 | 8.0E-04 | 2.0E-06 | 6.9E-07 | 381 | 0.233 |  |
| Stearoyl ethanolamide | C00013 | 7.2E-06 | 1.4E-06 | 8.7E-06 | 4.9E-06 | 0.8 | 0.656 |  |
| Stigmasterol | C00022 | N.A. | N.A. | 4.0E-06 | 1.2E-06 | <1 | N.A. |  |
| Succinic acid | No ID | 3.4E-04 | N.A. | 6.9E-04 | 1.5E-04 | 0.5 | N.A. |  |
| Sulfotyrosine | No ID | 8.9E-05 | N.A. | N.A. | N.A. | 1< | N.A. |  |
| Taurine | C08365 | 3.4E-03 | 3.7E-04 | 6.6E-03 | 2.9E-04 | 0.5 | 4.3E-04 | *** |
| Taurochenodeoxycholic acid | No ID | 2.1E-04 | 7.2E-05 | 4.3E-06 | 3.9E-06 | 49 | 0.039 | * |
| Taurocholic acid | C03172 | 1.6E-03 | 1.1E-03 | 2.2E-05 | 2.9E-05 | 71 | 0.137 |  |
| Taurodeoxycholic acid | C00213 | 2.7E-06 | 6.6E-07 | 2.5E-05 | 3.2E-05 | 0.11 | 0.349 |  |
| Taurolithocholic acid | No ID | 1.0E-06 | N.A. | 6.4E-06 | N.A. | 0.2 | N.A. |  |
| Tauroursodeoxycholic acid | C00065 | N.A. | N.A. | 1.2E-06 | N.A. | <1 | N.A. |  |
| Terephthalic acid | C01753 | 2.8E-04 | 4.0E-05 | 2.9E-04 | 3.0E-05 | 1.0 | 0.901 |  |
| Thiamine | C00836 | 4.5E-05 | 2.7E-05 | N.A. | N.A. | 1< | N.A. |  |
| Thiaproline | C00319 | 3.2E-05 | N.A. | 9.3E-05 | 6.1E-06 | 0.3 | N.A. |  |
| Thr | C06124 | 2.3E-02 | 1.0E-03 | 4.6E-02 | 1.7E-03 | 0.5 | 1.5E-04 | *** |
| Threonic acid | C10172 | 9.5E-04 | 5.6E-04 | 9.6E-04 | 7.3E-05 | 1.0 | 0.991 |  |
| Thyroxine | C01530 | 3.7E-06 | 5.9E-07 | N.A. | N.A. | 1< | N.A. |  |
| Triethanolamine | C16300 | 3.0E-05 | 1.3E-06 | 3.2E-05 | 7.3E-06 | 0.9 | 0.679 |  |
| Trigonelline | No ID | 4.9E-05 | 1.9E-05 | 5.2E-04 | 1.8E-04 | 0.09 | 0.043 | * |
| Trimethylamine *N*-oxide | C05442 | 5.0E-03 | 3.1E-03 | 8.6E-04 | 2.9E-04 | 5.8 | 0.143 |  |
| Trp | C00042 | 1.9E-02 | 4.3E-03 | 2.5E-02 | 1.5E-03 | 0.8 | 0.128 |  |
| Tyr | No ID | 1.3E-02 | 2.3E-04 | 9.3E-03 | 5.8E-04 | 1.4 | 0.005 | ** |
| Urea | C00245 | 8.5E-01 | 9.3E-02 | 2.0E-01 | 4.2E-02 | 4.3 | 0.002 | ** |
| Uric acid | C05465 | 4.0E-04 | 1.4E-04 | 8.1E-04 | 1.2E-04 | 0.5 | 0.020 | * |
| Uridine | C05122 | 6.1E-04 | 1.3E-04 | 5.0E-04 | 4.8E-05 | 1.2 | 0.259 |  |
| Urocanic acid | C05463 | N.A. | N.A. | 7.6E-05 | 7.7E-06 | <1 | N.A. |  |
| Ursodeoxycholic acid | C02592 | N.A. | N.A. | 1.8E-06 | 1.1E-06 | <1 | N.A. |  |
| Val | No ID | 7.6E-02 | 8.3E-03 | 4.7E-02 | 2.8E-03 | 1.6 | 0.018 | * |
| XA0002 | C06337 | 6.0E-04 | N.A. | N.A. | N.A. | 1< | N.A. |  |
| XA0003 | C00378 | N.A. | N.A. | 1.6E-04 | 2.1E-05 | <1 | N.A. |  |
| XA0012 | No ID | 8.4E-04 | 7.3E-05 | 1.0E-04 | N.A. | 8.2 | N.A. |  |
| XA0013 | C00188 | 1.7E-04 | N.A. | 1.6E-03 | 6.4E-04 | 0.11 | N.A. |  |
| XA0019 | C01620 | 5.0E-04 | 7.1E-05 | 4.1E-04 | 3.7E-05 | 1.2 | 0.294 |  |
| XA0027 | C01829 | 3.6E-03 | 2.1E-03 | 1.1E-03 | 8.3E-05 | 3.3 | 0.165 |  |
| XA0035 | C06771 | 2.1E-04 | 9.7E-05 | 2.7E-04 | 3.8E-05 | 0.8 | 0.570 |  |
| XC0016 | C01004 | 4.4E-04 | 1.3E-04 | 1.0E-03 | 1.8E-04 | 0.4 | 0.014 | * |
| XC0029 | C01104 | 3.9E-05 | 1.0E-06 | N.A. | N.A. | 1< | N.A. |  |
| XC0040 | C00078 | N.A. | N.A. | 6.6E-05 | 1.3E-05 | <1 | N.A. |  |
| XC0065 | C00082 | 7.1E-05 | 9.8E-06 | 5.8E-05 | 1.3E-05 | 1.2 | 0.275 |  |
| XC0120 | C00086 | 3.3E-04 | 7.2E-05 | 1.0E-03 | 1.1E-04 | 0.3 | 0.001 | ** |
| XC0132 | C00366 | 2.8E-05 | N.A. | N.A. | N.A. | 1< | N.A. |  |
| α-Tocopherol | C00299 | 1.5E-03 | 4.1E-04 | 1.1E-03 | 1.1E-04 | 1.4 | 0.223 |  |
| α-Tocopherol acetate-1 | C00785 | 2.0E-06 | N.A. | 9.5E-07 | N.A. | 2.1 | N.A. |  |
| α-Tocopherol acetate-2 | C07880 | 8.6E-06 | 5.8E-06 | 2.0E-06 | 5.9E-07 | 4.4 | 0.352 |  |
| β-Ala | C00183 | 7.0E-04 | 1.6E-04 | 1.7E-04 | 8.7E-06 | 4.1 | 0.028 | * |
| γ-Butyrobetaine | No ID | 4.0E-04 | 8.8E-05 | 2.3E-04 | 3.6E-05 | 1.7 | 0.065 |  |
| γ-Tocopherol | No ID | 1.8E-06 | N.A. | 7.0E-05 | 2.1E-05 | 0.03 | N.A. |  |
